# Strain-based discoordination imaging during exercise in heart failure with reduced ejection fraction: Feasibility and reproducibility

**DOI:** 10.1186/s12872-022-02578-w

**Published:** 2022-03-25

**Authors:** Louis S. Fixsen, Philippe C. Wouters, Richard G. P. Lopata, Hareld M. C. Kemps

**Affiliations:** 1 Department of Biomedical Engineering, Eindhoven University of Technology, P.O. Box 513, 5600 MB Eindhoven, The Netherlands; 2 Department of Cardiology, University Medical Centre Utrecht, Utrecht, The Netherlands; 3 Department of Cardiology, Maxima Medical Centre, Veldhoven, The Netherlands; 4 Department of Industrial Design, Eindhoven University of Technology, Eindhoven, The Netherlands

**Keywords:** Echocardiography, Heart failure, Cardiac resynchronization therapy, Exercise, Feasibility, Systolic rebound stretch

## Abstract

**Purpose:**

Various parameters of mechanical dyssynchrony have been proposed to improve patient selection criteria for cardiac resynchronization therapy, but sensitivity and specificity are lacking. However, echocardiographic parameters are consistently investigated at rest, whereas heart failure (HF) symptoms predominately manifest during submaximal exertion. Although strain-based predictors of response are promising, feasibility and reproducibility during exercise has yet to be demonstrated.

**Methods:**

Speckle-tracking echocardiography was performed in patients with HF at two separate visits. Echocardiography was performed at rest, during various exercise intensity levels, and during recovery from exercise. Systolic rebound stretch of the septum (SRSsept), systolic shortening, and septal discoordination index (SDI) were calculated.

**Results:**

Echocardiography was feasible in about 70–80% of all examinations performed during exercise. Of these acquired views, 84% of the cine-loops were suitable for analysis of strain-based mechanical dyssynchrony. Test–retest variability and intra- and inter-operator reproducibility at 30% and 60% of the ventilatory threshold (VT) were about 2.5%. SDI improved in the majority of patients at 30% and 60% of the VT, with moderate to good agreement between both intensity levels.

**Conclusion:**

Although various challenges remain, exercise echocardiography with strain analysis appears to be feasible in the majority of patients with dyssynchronous heart failure. Inter- and intra-observer agreement of SRSsept and SDI up to 60% of the VT were comparable to resting values. During exercise, the extent of SDI was variable, suggesting a heterogeneous response to exercise. Further research is warranted to establish its clinical significance.

## Introduction

For over 20 years, cardiac resynchronization therapy (CRT) has been an established device therapy for patients with heart failure (HF) with depressed left ventricular (LV) ejection fraction and LV conduction delay [1]. In addition to an electrical substrate amendable to CRT, patients that benefit from resynchronization are typically characterised by signs of mechanical dyssynchrony as well [2].

Echocardiographic assessment of such parameters has been increasingly investigated as a method to better screen patients eligible for CRT [3–5]. Nonetheless, randomised clinical trials that implemented parameters of mechanical dyssynchrony have thus far shown insufficient predictive ability to actually affect clinical decision making [6]. Consequently, clinical and echocardiographic response to CRT remains suboptimal in 30–40% of patients [7, 8].

There are various potential explanations for the lacking sensitivity and specificity of echocardiographic measures of mechanical dyssynchrony. First, parameters that were investigated prospectively often assessed relative timing differences within the LV [6, 9]. Instead, analysis of deformation characteristics (i.e. discoordination in LV strain) may better reflect myocardial work inefficiency [10, 11]. Second, echocardiographic evaluation of dyssynchrony is almost without exception performed at rest [6]. This is somewhat surprising, since patients with HF predominantly experience symptoms during exercise as a consequence of failing compensatory mechanisms. Mechanical dyssynchrony may therefore be exercise-dependent as well. Previous research has shown that echocardiographic parameters at rest do not necessarily represent (dys)synchrony during exercise [6, 12, 13]. Dyssynchrony during exercise may therefore be a superior predictor of response to CRT than parameters measured at rest alone [14–16]. However, to date, strain-based discoordination parameters have not yet been investigated.

Systolic rebound stretch of the septum (SRSsept), and acute restoration thereof, has been demonstrated as a strong predictor of response to CRT before [3, 17, 18]. SRSsept is a promising deformation-based parameter because it reflects myocardial work inefficiency within the septum [19]. SRSsept has been validated both in both computer models and patients, and may better reflect the mechanical substrate that is amenable to CRT than other indices of mechanical dyssynchrony [20].

For example, dyssynchrony can be overlooked at rest (i.e. “exercise-induced unmasking”) or disappear during exertion. To date, estimation of exercise-dependent discoordination using a strain-based parameter and its potential value has not been investigated. This is in part because assessment of SRSsept during exercise is challenging and faces a number of confounding factors (e.g. body motion and increased respiration, blood pressure and heart rate) that will affect the accuracy and interpretation of these measurements. The present exploratory study sought to investigate the feasibility and reproducibility [21] of SRSsept during exercise.

We determined the feasibility, test–retest variability, and the inter- and intra-observer reproducibility of SRSsept in heart failure patients during exercise tests. In addition, we evaluated potential exercise-induced unmasking or disappearance of SRSsept. Patients performed the exercise tests during two separate visits such that the reproducibility of the measurements could be determined. Additionally, variations in SRSsept during exercise were explored.

## Methods

Patients (n = 18) with stable CHF that attended the outpatient clinic of the Máxima Medical Centre (Veldhoven, The Netherlands) were prospectively included. All patients provided written informed consent. The study protocol conformed to the principles outlined in the Declaration of Helsinki on research in human subjects and to the procedures of the regional Medical Ethics Committee. All experimental protocols were approved by the Institutional Review Board of Maxima Medical Center (Veldhoven, the Netherlands).

Patients included in the study must have HF with an LVEF ≤ 35%. Patients were excluded if they had: a myocardial infarction or unstable angina less than 3 months prior to inclusion; any diseases (orthopaedic, vascular, pulmonary or neuromuscular) that limited exercise capacity such that exercise tests were not feasible; clinical signs of decompensated heart failure; documented ventricular tachycardia or ischemia during exercise; intra-cardiac shunts or congenital heart disease limiting exercise capacity.

Each patient made three visits to the hospital. The first visit consisted of a routine clinical examination and incremental (symptom limited) exercise test with respiratory gas analysis in order to determine the ventilatory threshold (VT). The VT was used to determine exercise intensity of the submaximal exercise performed in visits 2 and 3. Exercise echocardiography was performed by the same sonographer during visit 2 and 3. Ultrasound data were also acquired before (i.e. rest) and after (i.e. recovery) the exercise bouts.

### Symptom limited incremental exercise testing

After the initial clinical examination, a symptom limited, incremental exercise test with respiratory gas analysis was performed on a cycle ergometer (Lode Corival, Groningen, The Netherlands), using an individualised ramp protocol aiming at a total test duration of 8–12 min; Patients were instructed to maintain a pedalling frequency of 70 rotations per minute. A twelve-lead electrocardiogram (ECG) was recorded continuously. Peak oxygen uptake (peak VO_2_) was defined as the average value of oxygen uptake during the last 15 s of exercise. VT was assessed by the V-slope method [22]. If symptom limited exercise test had already been performed within the previous 3 months, these data were used to establish the exercise protocol for exercise echocardiography.

### Exercise echocardiography measurements

**Fig. 1:**
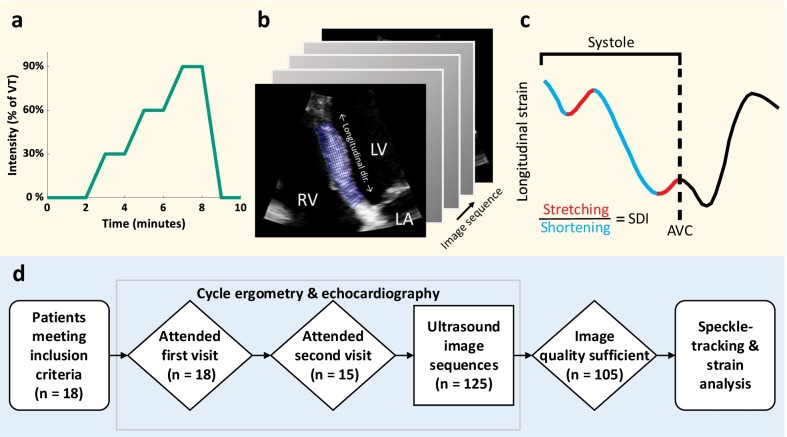
Study overview. **a** Heart failure patients were subjected to echocardiography during exercise at various intensity levels of the ventilatory threshold (VT). **b**, **c** This was followed by segmentation and and automated analyses of septal systolic rebound stretch (SRSsept). **d** Flowchart of ultrasound data processing; from cycle ergometry to speckle tracking-based strain analysis. Legend: *AVC* aortic valve closure, *LA* left atrium, *LV* left ventricle, *RV* right ventricle, *SDI* septal discoordination index, *SRSsept* septal rebound stretch of the septum, *VT* ventilatory threshold

The exercise protocol was performed on an Echo Cardiac Stress table (Lode, Groningen, The Netherlands). Patients were placed in a supine position and rotated an additional 45 degrees around the longitudinal axis. The exercise protocol was constructed from the results of the symptom limited incremental exercise test with respiratory gas analysis and consisted of three 2-min exercise bouts, respectively at 30%, 60%, 90% of the VT (Fig. 1a).

Two-dimensional ultrasound DICOM cine-loops were acquired with a Philips Epiq 7C scanner (Philips Healthcare, Best, The Netherlands) equipped with an X5-1 transducer. The cine-loops had a resolution of 800 by 600 pixels and a frame rate of 50 to 90 frames per second. For the septal strain analysis, zoomed-in (i.e., narrow aperture), high frame rate (90 Hz) cine-loops of the inter-ventricular septum were acquired (Fig. 1b). LV end-diastolic and end-systolic volumes and ejection fraction were assessed by the biplane Simpson’s disk method. Mitral valve closure (MVC) and aortic valve closure (AVC) were determined at rest from Doppler flow measurements. Where Doppler measurements were unavailable these were determined visually from parasternal long-axis cine-loops.

Data were excluded for image quality reasons if there was an incomplete view of the septum through the entire cardiac cycle; either due to significant image artefacts, out-of-plane motion of the heart, or structures preventing transmission of ultrasound.

Systolic and diastolic times vary non-linearly with changes in heart rate from rest [23]. Timing of AVC and cardiac cycle length were therefore scaled to account for changes in the ratio between systolic and diastolic times relative to rest, using data from Bombardini et al. [23]. Systolic and diastolic times during stress and relaxation at the provided heart rates were fitted to a curve with smoothing splines. Each patient’s measured heart rate during exercise and recovery was then evaluated along the fitted lines, yielding an estimate of expected change in systolic and diastolic time.

### Speckle tracking

Speckle tracking was performed on DICOM image sequences using a custom strain imaging toolbox implemented in MATLAB (revision 2019b, 64-bit, The Mathworks Inc. Natick, MA, USA), previously described in detail by Lopata et al. [24]. The septum was segmented from the closest visible portion in the direction of the apex until the level of the mitral and tricuspid valves, or the closest visible point distal of the valves (Fig. 1b). Segmentation of the septum was performed by two independent observers (LF and PW) who were blinded to outcome (i.e. resting/exercise phase). A mesh of 11 radial by 31 longitudinal points was generated over the segmented area, obeying a local coordinate system. A 2-D block matching algorithm was then used to estimate inter-frame displacements of different regions of pixels and mapped to the intersecting points of the mesh. Cumulative strain in the longitudinal direction was calculated with a least-squares strain estimator, taking the spatial derivative of the deformation of the mesh from the initially segmented configuration. The resulting strain field over the visible septal region was then averaged, yielding septal longitudinal strain over time.

### Mechanical discoordination indices

The determination of SRSsept has previously been described in detail by De Boeck et al. [3]. In short, average deformation throughout the visible septum (blue segmented area in Fig. 1b) at each time point was used to create curves of longitudinal strain over time. Longitudinal strain within the systolic period was grouped into shortening and stretching components (Fig. 1c, blue and red portions respectively). SRSsept as an index of *wasted* septal work was thereafter calculated as the sum of systolic stretch within the septum that occurs directly after premature termination of longitudinal shortening. Conversely, systolic shortening (SS) was defined as the absolute sum of shortening during systole, a measure of *effective* work performed. Increased blood pressure during exercise exertion typically leads to a reduction in the overall magnitude of systolic strains, therefore complicating the comparison of systolic strain magnitudes during exercise. We therefore calculated the *ratio* between SRSsept and SS, here referred to as the septal discoordination index (SDI) [25], as shown in Fig. 1c. This yields a measure of relative wasted and constructive work in the septum [26].

### Statistical analysis

The similarity of the septal longitudinal strain curves (i.e. cumulative strain in the longitudinal direction) from each visit was assessed by Pearson’s correlation at each exercise intensity level. Prior to correlation, the strain curves were normalised both in amplitude and number of samples (i.e., if there was a discrepancy in heartrate between the two visits); longer cardiac cycles were down-sampled to match the length of the shorter cycle. Intra- and inter-observer agreement was assessed through Bland–Altman analysis. Limits of agreement are given in absolute strain values. Continuous variables are presented as means with standard deviation and dichotomous data as percentages. The paired Student’s t-test was used to assess differences between consecutive measurements. A significance level of 0.05 was used. The test–retest, intra-observer, and inter-observer reliability of SRSsept was assessed by calculating the intraclass correlation coefficient (ICC), using a two-way mixed effects model to determine the absolute agreement of single measurements. Agreement between exercise-induced changes in SDI at different exercise intensity levels was assessed using Cohen's kappa coefficient. All data were analysed in MATLAB.

## Results

### Feasibility

Of the 18 patients that were included and attended the first echocardiographic visit, 15 patients also attended the second visit (median 7 days apart; 165 total possible cine-loops). All but one patient were male, and half of the patients had an ischemic cardiomyopathy (Table 1). Ultimately, 137 cine-loops were acquired (83% of potential loops). As such, in 88% of the 33 distinct patient visits at least 2 out of 3 sequences during exercise were successfully acquired. Of the acquired cine-loops, 20 loops were excluded due to insufficient image quality and 12 sequences due to the patient only attending one visit. Therefore, 105 image sequences (77% of acquired loops) remained for strain analysis, in 12 of the 18 patients (Fig. 1d).

**Table 1:** Characteristics of the cohort included in this study

	Total (n = 18)	Paired data (n = 15)
General characteristics		
Male gender	17 (94%)	14 (93%)
Age (yr)	67 (59–74)	71 (64–75)
BMI (kg/m^2^)	26.2 (23.2–30.7)	24.7 (22.6–30.6)
NYHA I	4 (22%)	4 (27%)
NYHA II	8 (44%)	7 (46%)
NYHA III	6 (33%)	4 (27%)
Comorbidities		
Ischemic cardiomyopathy	9 (50%)	6 (40%)
Medication		
Beta blockers	17 (94%)	14 (93%)
ACE-inhibitors/Angiotensin II receptor blockers	18 (100%)	15 (100%)
Diuretics	14 (78%)	12 (80%)
Aldosterone antagonists	7 (39%)	5 (33%)
ECG characteristics		
Sinus rhythm	18 (100%)	15 (100%)
QRS duration (ms)	131 ± 30.1	129 ± 29
QRS duration ≥ 130 (ms)	10 (56%)	8 (53%)
LBBB	8 (44%)	6 (40%)
Lab results		
NT-proBNP (pmol/l)	125 (67–205)	125 (67–205)
Echocardiography		
End-diastolic volume (ml)	174 (148–231)	171 (144–225)
End-systolic volume (ml)	124 (96.4–177)	121 (96–173)
Ejection fraction (%)	28.6 (26.5–33.1)	29.0 (24.3–33.3)

*BMI* body mass index, *NYHA* New York Heart Association, *ACE* Angiotensin-converting enzyme, *ARB* Angiotensin II receptor blockers, *ECG* electrocardiogram, *LBBB* left bundle branch block, *NT-proBNP* N-terminal prohormone of brain natriuretic peptide

Table 2 shows an overview of the data included in the strain analysis. The remaining loops were graded at each measurement point (baseline, 30%, 60% and 90% of VT, and recovery) in terms of image quality (good, moderate and poor). Grading was based on the image contrast, definition of tissue structures, image plane stability, and presence of artefacts (or lack thereof). The number of cine-loops of a poor quality increased from 12 to 42% between rest (baseline) and high intensity exercise (90% VT). The mean length of ejection (during which SRSsept was calculated) was 0.34 ± 0.05 s, resulting in an average of 30.7 ± 4.6 frames during ejection.

**Table 2:** Summary of patient heart rate (median and interquartile range) and ultrasound data acquired at each intensity that were included in the final strain analysis

	Baseline	30% VT	60% VT	90% VT	Recovery
Heart rate					
Visit 1	60 (55.3–63)	79 (68–83)	90 (77.8–94.3)	97 (80.5–106)	78 (70–95.5)
Visit 2	63 (55–68.8)	76 (70–83)	85 (78.5–89.75)	94 (87–102)	88 (73.5–92)
Number					
Acquired	24	30	27	27	29
Attended twice	22 (91.7%)	25 (83.3%)	26 (96.3%)	24 (88.9%)	28 (96.6%)
Sufficient quality	20 (83.3%)	20 (66.7%)	22 (81.5%)	19 (70.4%)	24 (82.8%)
Image quality					
Good	9 (45%)	10 (50%)	8 (36.4%)	3 (15.8%)	8 (33.3%)
Moderate	9 (45%)	9 (45%)	10 (45.5%)	9 (47.4%)	8 (33.3%)
Poor	2 (10%)	1 (5%)	4 (18.2%)	7 (36.8%)	8 (33.3%)

*VT* ventilatory threshold

### Agreement and variability

**Fig. 2:**
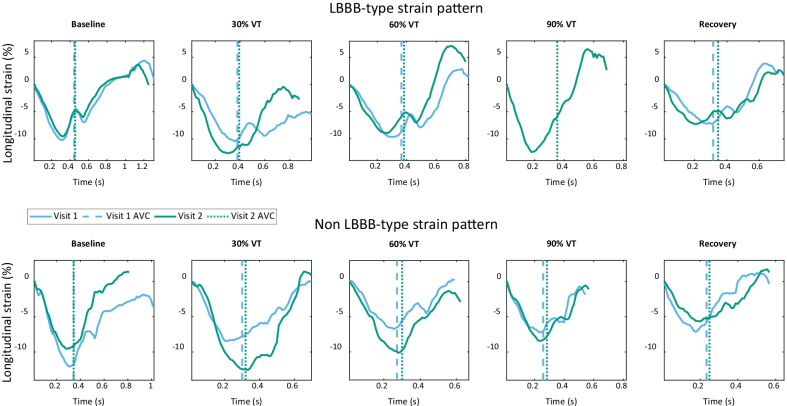
Septal strain curves in a patient with an LBBB-type strain pattern (upper), as seen in [20], and a patient with non LBBB-type strain pattern (lower), during each exercise intensity. Curves begin at mitral valve closure, with aortic valve closure (AVC) marked by dotted and dashed lines. Blue lines show strain during visit 1, green visit 2

Longitudinal strain was calculated in the septal region of the acquired ultrasound cine-loops (Fig. 2). In the first visit, prior to 90% VT, the patient with LBBB-type strain ceased the exercise protocol. There was generally good strain curve similarity between visits, with some differences in overall magnitude and period between tests. Median correlation (baseline, 0.91; 30%, 0.81; 60%, 0.86; 90%, 0.89; recovery, 0.86) ranged between 0.81 in the worst case (30% of VT) and 0.91 in the best case (baseline). However, interquartile ranges of correlation increased greatly between the baseline measurement and all further intensities. There was no significant correlation between image quality grade and strain curve similarity.

**Fig. 3:**
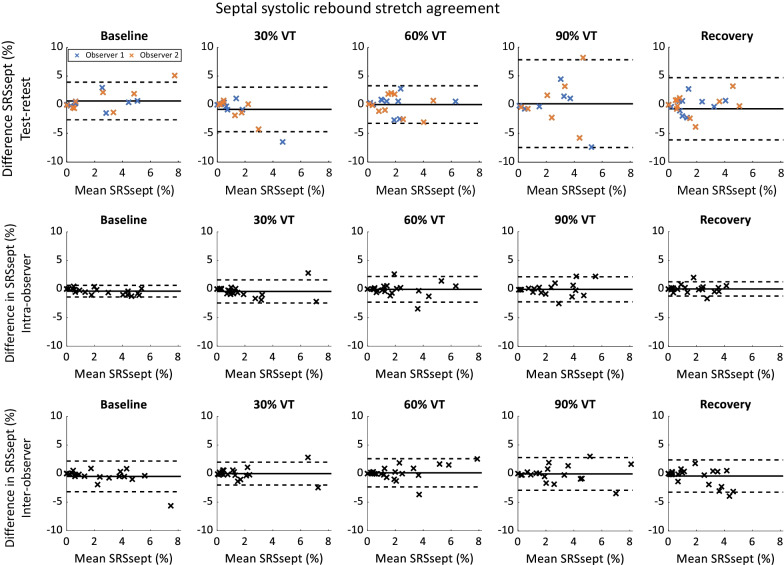
Bland–Altman plots showing test–retest (upper), intra-observer (middle) and inter-observer (lower) differences in septal systolic rebound stretch (SRSsept). Different colour denotes observer (upper only)

Bland–Altman plots showing the test–retest agreement of SRSsept are shown in Fig. 3 (upper row). Agreement for SRSsept at 30% and 60% of VT was comparable to agreement at baseline, with differences in SRSsept below 5% (mean ± standard deviation: baseline, 0.66 ± 1.67%; 30% VT, − 0.81 ± 1.98%; 60% VT, 0.03 ± 1.67%). During higher exercise intensity (specifically 90%) and recovery, the confidence interval was wider and measurements with a large bias between visits were present.@@@

Intra-observer limits of agreement for SRSsept were lowest at baseline (mean − 0.37 ± 0.52%), and consistently increased when exercise continued to intensify. Limits of agreement remained within 2.3% at all exercise intensities and during recovery (Fig. 3, middle row). Conversely, inter-observer limits of agreement for SRSsept were within 2.8% at baseline (mean − 0.48 ± 1.37%) and remained similar for all intensities (Fig. 3, lower row).

**Fig. 4:**
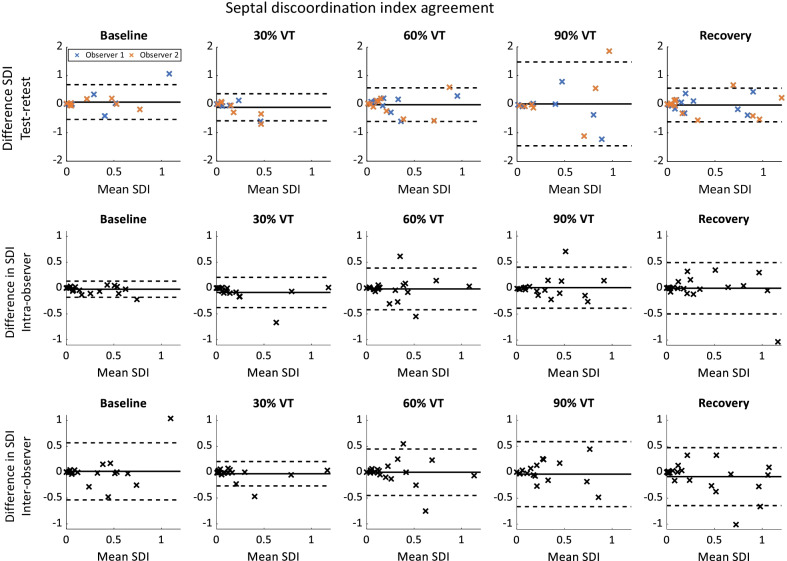
Bland–Altman plots showing test–retest (upper), intra-observer (middle) and inter-observer (lower) differences in septal discoordination index (SDI). Different colour denotes observer (upper only)

Test–retest agreement for SDI (Fig. 4, upper row) was comparable at rest, 30% and 60% of VT and in recovery (baseline, 0.07 ± 0.31; 30% VT, − 0.10 ± 0.24; 60% VT, − 0.02 ± 0.30; recovery, 0.03 ± 0.30). However, at 90% VT agreement was poor (0.01 ± 0.75). Intra-observer agreement of SDI (Fig. 4, middle row) during exercise far exceeded the variability at baseline (baseline, − 0.02 ± 0.08) but remained similar during exercise and recovery, and was comparable to baseline test-rest agreement. Likewise, inter-observer agreement of SDI was comparable to baseline test–retest agreement, other than at 30% of VT where the variability was lower (0.03 ± 0.12).

### Reliability

Intraclass correlation coefficients (ICCs) for test–retest, intra-observer, and inter-observer reliability of SRSsept are shown in Table 3. Test–retest reliability was highest for baseline measurements and at 60% of VT (ICCs = 0.82 and 0.66, respectively). There was poor test–retest reliability of SRSsept at 30%, 90%, and in recovery (ICCs = 0.23, 0.04 and 0.27 respectively). Intra- and inter-observer reliability was good at all exercise intensities (ICC ≥ 0.86 and ICC ≥ 0.80, respectively).

**Table 3:** Intraclass correlation coefficients and coefficient of variation of strain parameters, septal systolic rebound stretch (SRSsept), and the septal discoordination index (SDI) at each exercise intensity

	Baseline	30% VT	60% VT	90% VT	Recovery
SRSsept					
Intraclass correlation coefficient					
Test–retest	0.82	0.23	0.66	0.04	0.27
Intra-observer	0.95	0.86	0.88	0.89	0.97
Inter-observer	0.80	0.88	0.83	0.82	0.86
SDI					
Intraclass correlation coefficient					
Test–retest	0.50	0.32	0.64	0.16	0.77
Intra-observer	0.98	0.86	0.74	0.87	0.80
Inter-observer	0.66	0.92	0.72	0.76	0.75

SDI shows poor test–retest reliability at 30% and 90% of VT (ICC = 0.32 and 0.16, respectively). Reliability is moderate to good elsewhere (ICCs = 0.50 to 0.77). Intra-observer reliability of SDI is good (ICC > 0.74) for all intensities. Inter-observer reliability of SDI is lowest at baseline (ICC = 0.66).

### Exercise induced changes in septal systolic rebound stretch

There were 15 distinct sets of cine-loops that contained examinations performed at rest and at 30% and 60% of the VT (i.e., the reproducible exercise intensities). Exercise-induced changes in SDI relative to rest were variable, since consistent improvement (n = 8), consistent worsening (n = 4) or a reciprocal response (n = 3) were observed. Agreement (i.e. relative improvement versus relative worsening) between exercise-induced ∆SDI at 30% and 60% of the VT were moderate to good (Cohen K = 0.57 and 0.73 for observer 1 and 2 respectively).

**Fig. 5:**
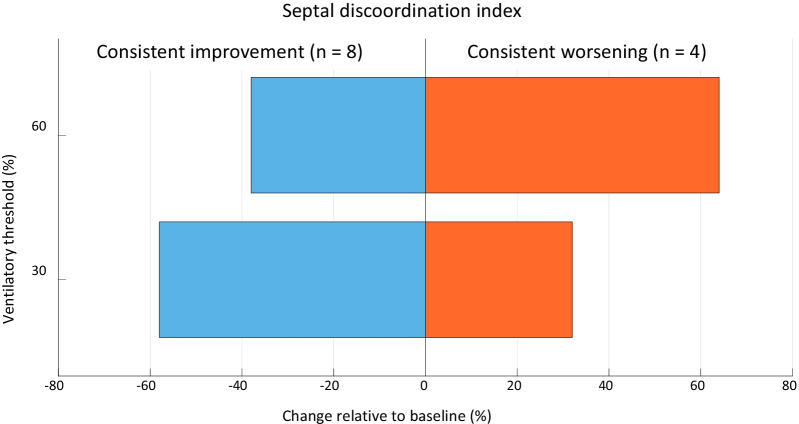
Heterogeneous effect of exercise on septal discoordination index. Relative to rest, exercise at 30% and 60% of the ventilatory threshold elicits either a consistent improvement (blue) or consistent further deterioration of septal coordination (orange)

Relative to baseline, the majority of patients demonstrated an improvement in mechanical coordination at 30% and 60% of the VT, with on average a 58% or 38% reduction in SDI, respectively. Conversely, a 32% and 64% increase in SDI was seen in the cases with consistent exercise-induced deterioration of strain-efficiency at 30% and 60% of the VT respectively (Fig. 5).

## Discussion

Our findings show that measurement of longitudinal strain of the septum (i.e. systolic rebound stretch and systolic shortening) during exercise stress tests is challenging but feasible (84% feasibility in obtained image sequences). In addition, SRSsept during exercise has a reproducibility comparable to that of measurements performed at rest, up to a moderate (60% of ventilatory threshold) level of exercise, using the present protocol. We were able to identify heterogeneous exercise-induced alterations in SDI relative to rest, which were in moderate to good agreement at both intensity levels.

### Feasibility

We report a feasibility of 84% for measuring strain during exercise when cine-loops could be acquired successfully. Where cine-loops could not be acquired, reasons for this were likely inherent to the complexity and demandingness of performing echocardiography at multiple exercise intensity levels and/or patients prematurely ceasing the exercise protocol. Exercise stress-tests introduce several confounding elements (e.g. greater body motion and increased respiration) into otherwise routine echocardiographic measurements, thereby significantly affecting image quality.

Temporal consistency of the image plane is vital in speckle tracking analysis, since strain is calculated based on the inter-frame displacement of pixels. Complete or partial occlusion, or out-of-plane motion of the region-of-interest during tracking causes decorrelation during displacement estimation. This in turn has a significant negative effect on the accuracy of strain estimation. The image quality criteria we deemed necessary for accurate displacement tracking were therefore stricter than those that were sufficient for measurement of end-systolic and diastolic volumes.

### Test–retest reproducibility and reliability

Resting test–retest septal strain curve correlation was excellent and comparable to previous studies concerning SRSsept measured at rest [27]. During exercise at 30% and 60% of the VT and during post-exercise recovery, test–retest variability of SRSsept was comparable to baseline. Our results therefore suggest adequate agreement of SRSsept between two separate visits.

These findings are in line with previous research that measured segmental longitudinal strain of the LV [28]. Here, depending on the commercial vendor, an absolute difference of up to 5% between first and second image acquisition was demonstrated. Some variability is always expected due to measurement variability, differences in the acquired acoustic window, and physiological fluctuations that occur over time. Because the present study performed echocardiographic examinations on two separate days, more pronounced physiological variation is to be expected (and therefore a lower measurement repeatability) when compared to research that performed both examinations at most two hours apart, thereby increasing our test–retest variability [28, 29]. Similarly, poor test–retest reliability is shown in the SRSsept ICCs, which was in contrast to the relatively good ICC for intra-observer and inter-observer.

### Intra- and interobserver reproducibility

As expected, intra- and inter-observer reproducibility was better than test–retest agreement, owed primarily to the lack of physiological variation in these measurements. We acknowledge that variability of 2.8% for SRSsept between different observers is likely to be clinically relevant in regard to predicting response to CRT. Importantly however, because repeated analyses of identical image datasets were performed using the same software, inter-observer variability can solely be attributed to differences in either cycle-selection or segmentation. Variability may therefore be inherent to regional STE-based strain-analysis.

In line with these findings, similar variability (i.e. 1.0 ± 2.0%) of SRSsept between different vendors was found by Van Everdingen et al., resulting in varying C-statistic values and different cut-off values [27]. Since a variability of up to 5% can be expected when measuring segmental strain [28], it is reasonable to suspect that, perhaps, similar amounts of variability were present in studies that investigated SRSsept (at rest) in a clinical setting [17, 18]. Importantly, these studies have already proven the importance of SRSsept in the selection of patients with HF eligible for CRT, regardless of the underlying variability there. Future studies, with larger sample sizes, are warranted to establish more accurate estimates of variability for SRSsept during exercise, and directly compare these values to SRSsept at rest.

### Exercise-dependent mechanical (dis)coordination in heart failure

In almost all patients, pronounced but variable exercise-induced changes in SDI were observed. These findings agree with previous research [12, 15] that investigated timing-based parameters of dyssynchrony. Although exercise-induced response was heterogeneous (i.e. either improved or worsening of septal coordination), the interpretation at either exercise intensity level (i.e. 30% and 60% of VT) was consistent with moderate to good agreement.

Importantly, SDI reflects the *ratio* between wasted and constructive systolic strain of the septum during systole. SDI therefore allows for better inter- and intra-individual comparison at various exercise intensity levels than SRSsept alone, which is more sensitive to changes in blood pressure [30]. Changes in SDI during exertion may therefore indicate (lack of) cardiac compensation mechanisms during mild to moderate exertion. The heterogeneity of exercise-induced changes in SDI may therefore in part explain the vast range of symptom-severity many patients with HF experience during daily activities, despite having similar echocardiographic function at rest.

In line with this hypothesis, previous research demonstrated the association between (the degree of) exercise-induced improvement of mechanical dyssynchrony and non-response to CRT [14, 15]. Whether this also holds true for strain-based indices of discoordination is unknown. Therefore, further investigation is required in a larger cohort to ascertain the clinical utility of these measurements.

### Limitations and future work

Since the goal of the present study is hypothesis-generating, the preliminary nature of our findings must be acknowledged, and our results should be interpreted accordingly. First, cycle ergometry measurements were performed several days apart because of the exertion patients had to undergo, increasing physiological variation and thereby reducing test–retest reliability compared to other studies. Although ‘zoomed’ high frame-rate ultrasound allowed for high quality imaging of the septum, the narrow aperture prevented capture of the whole septum in some cases. Whilst the practical benefits of SRSsept as a deformation parameter during exercise are clear, it is limited by its high variability, which are largely inherent to regional strain indices. Sample size in this explorative study was too low in order to produce a meaningful coefficient of variation (i.e. accurately reflecting the corresponding population with a sufficiently narrow confidence interval) and were therefore not calculated.

The poor image quality caused by breathing and body movement during exercise meant that measurement of consecutive cardiac cycles was not possible. Often only a single cycle was of a sufficient quality for strain analysis at a given measurement point. Because of the difficulty of acquiring pulsed-wave Doppler of the aortic valve during exercise, AVC had to be scaled based on estimated systolic and diastolic times for a given heart rate during exercise. This reduced the accuracy of SRSsept measurements and lead to an error of unknown magnitude for both systolic shortening and SRSsept. This is a significant limitation of the present study. The lack of simultaneous blood pressure measurements throughout the exercise protocol may hamper clinical understanding of our results. Finally, the strain analysis software used in this study was not standard commercially available software, however it has previously been compared to such software on clinical data where it was found to produce similar results [31].

Future studies should ensure that the exact timing of aortic valve closure is known, either assessed visually or with Doppler flow measurements. Improvements could be made to the protocol and quality of data by: use of full field-of-view imaging at a high (> 90 Hz) frame rate to allow measurement of strain over the complete septal region; inclusion of simultaneous blood pressure measurement throughout the exercise protocol; minimising time between measurement days to reduce physiological variation; and minimising the effects of exercise on image quality, for instance by use of an ultrasound probe fixation device.

## Conclusion

Measuring mechanical discoordination using STE during exercise is feasible when high frame-rate image acquisition with semi-automated and vendor-independent analysis is ensured, but various challenges remain. During exercise up to 60% of the ventilator threshold, both test–retest agreement and inter-operator variability remained comparable to measurements performed at rest, with moderate baseline SRSsept variability prior to exercise. Moreover, exercise may elicit either a consistent improvement or deterioration of septal coordination in patients with dyssynchronous heart failure. Although interesting, its potential clinical utility remains to be further explored in larger trials with CRT recipients.

## Data Availability

The datasets used and/or analysed during the current study are available from the corresponding author on reasonable request.
